# Frequency of ABO, Rh Subtypes, and Kell Antigen Among Blood Donors at Riyadh Regional Blood Bank: A Retrospective Cross-Sectional Study

**DOI:** 10.3390/jcm15166165

**Published:** 2026-08-08

**Authors:** Hisham Abdulrahman Aloshaywan, Rimah Abdullah Saleem, Muhammad Raihan Sajid, Hani Tamim, Salman Aldosari, Hibba Siraj, Anas M. Alkhabaz, Lara M. Samhan, Momo Arai, Abdulwahab Binjomah

**Affiliations:** 1 College of Medicine, Alfaisal University, Riyadh 11533, Saudi Arabiahsiraj@alfaisal.edu (H.S.);; 2 Central Blood Bank, Ministry of Health, Riyadh 12746, Saudi Arabia; 3 Department of Biochemistry and Molecular Medicine, College of Medicine, Alfaisal University, Riyadh 11533, Saudi Arabia; rsaleem@alfaisal.edu; 4 Department of Pathology, College of Medicine, Alfaisal University, Riyadh 11533, Saudi Arabia; 5 Department of Internal Medicine, Clinical Research Institute, American University of Beirut Medical Center, Beirut 1107, Lebanon; 6 Bacterial Diseases and Special Pathogens Department, Public Health Authority, Riyadh 13351, Saudi Arabia

**Keywords:** ABO blood group, Rh phenotype, Kell antigen, K antigen, blood donors, Saudi Arabia, transfusion medicine, blood group frequency, alloimmunization, ethnicity

## Abstract

**Background/Objectives:** Blood group antigen frequencies vary across ethnic populations and are critical for safe transfusion practice, inventory management, and alloimmunization prevention. This study aimed to determine ABO blood group, extended Rh phenotype (D, C, c, E, e), and K antigen frequencies among blood donors at the Riyadh Regional Blood Bank, and to examine their associations with donor ethnicity. **Methods:** This retrospective cross-sectional study analyzed 10,463 blood donors (January–December 2021) using the Immucor NEO Iris fully automated immunohematology analyzer. Donors were classified into five ethnic groups. Chi-square tests assessed associations (*p* < 0.05), the primary scientific contribution is the descriptive frequency data, with hypothesis testing presented as secondary and exploratory. **Results:** Most donors were male (95.2%). Blood group O+ was most prevalent (31.6%); AB− was rarest (0.9%). Rh D-positivity was 84.7%, lowest among Saudis (81.1%). The most frequent Rh phenotypes were CcDee (24.2%), ccDee (19.9%), and CCDee (17.7%). K antigen positivity was 13.7%. Significant associations were found between D antigen and ethnicity (*p* < 0.001), Rh phenotype and ethnicity (*p* = 0.003), and ABO and ethnicity (*p* < 0.001). A hypothesis-generating association between Rh phenotype and K antigen (*p* = 0.019) did not meet the Bonferroni-corrected threshold and is considered exploratory. **Conclusions:** This study provides, to our knowledge, one of the largest ethnically stratified blood group datasets from Riyadh to date. The high D-negativity among Saudi donors (18.9%) has direct implications for extended phenotyping protocols and rare donor registry development. The association between Rh phenotype and K antigen requires molecular confirmation before any biological or clinical conclusions can be drawn.

## 1. Introduction

Blood transfusion is one of the most frequently performed medical interventions worldwide, underpinning life-saving therapies across diverse clinical contexts, including trauma, surgical hemorrhage, oncology, and chronic hematological disorders [1]. Safe transfusion practice depends fundamentally on accurate blood group typing and on preventing incompatible red blood cell (RBC) transfusions, which can trigger potentially fatal hemolytic reactions. While ABO and D (Rh) typing are universal pre-transfusion requirements, extended antigen phenotyping—including Rh subtype antigens and the Kell system—is increasingly recognized as essential for high-risk populations, including patients with sickle cell disease and thalassemia [2].

Blood group antigens are polymorphic structures on the RBC surface whose inheritance is governed by specific gene loci. The International Society of Blood Transfusion (ISBT) recognizes 45 blood group systems comprising over 360 distinct antigens [3]. Among these, the ABO, Rh, and Kell systems are of paramount clinical importance due to their capacity to induce alloantibodies that mediate acute and delayed hemolytic transfusion reactions, and hemolytic disease of the fetus and newborn (HDFN) [4].

The ABO system consists of four principal blood groups—A, B, AB, and O—determined by the presence or absence of A and B antigens on the RBC surface [5]. ABO is unique in that naturally occurring antibodies against absent antigens are present from early infancy, making ABO incompatibility the leading cause of acute hemolytic transfusion reactions [6]. ABO antigens have also been associated with differential susceptibility to infectious diseases, coagulation factor levels, and cancer risk [5].

The Rh blood group system is the most polymorphic, encompassing 56 antigens encoded by two closely linked genes, *RHD* and *RHCE* [7]. The five principal antigens—D, C, c, E, and e—define the clinically relevant Rh phenotype. The D antigen is the most immunogenic minor blood group antigen: D-negative individuals exposed to D-positive RBCs have up to an 80% likelihood of developing anti-D alloantibodies [7]. Rh alloantibodies are major causes of delayed hemolytic transfusion reactions, severe HDFN, and alloimmunization in patients with sickle cell disease receiving chronic transfusions [8].

The Kell blood group system comprises approximately 35 antigens. The K antigen (KEL1) ranks second only to D in immunogenicity, with anti-K antibodies responsible for 15–20% of RBC alloimmunization during pregnancy [9]. Kell-mediated HDFN is particularly severe because anti-K antibodies suppress fetal erythropoiesis by targeting early erythroid progenitor cells, causing anemia disproportionate to antibody titers [9,10].

Blood group antigen frequencies vary significantly across ethnic and geographic populations, with important implications for inventory management, alloimmunization risk prediction, and availability of rare antigen-matched blood [11]. Saudi Arabia hosts one of the world’s most ethnically heterogeneous populations, with a large indigenous Saudi population coexisting with expatriates from over 50 nationalities [12]. Comprehensive data integrating extended Rh phenotyping, Kell antigen distribution, and cross-correlations with donor ethnicity in a single large Riyadh cohort remains lacking [13,14,15,16,17,18].

This study aimed to determine ABO blood group, extended Rh phenotype (all five principal antigens and 18 phenotypic combinations), and K antigen frequencies among blood donors at the Riyadh Regional Blood Bank (Riyadh, Saudi Arabia). We further examined associations between these blood group systems and the ethnic composition of the donor population to provide evidence-based guidance for transfusion medicine practice and rare donor registry development. The primary contribution of this work is the establishment of comprehensive baseline frequency data for this diverse donor population, which serves as a foundation for future molecular and patient-focused studies.

## 2. Materials and Methods

### 2.1. Study Design and Setting

This was a retrospective cross-sectional study conducted at the Riyadh Regional Blood Bank, a tertiary referral blood banking facility serving Greater Riyadh, Kingdom of Saudi Arabia, covering the period 1 January 2021 to 31 December 2021. Ethics approval was granted by the Institutional Review Board (IRB) of King Saud Medical City, Riyadh, Saudi Arabia (Reference: H1RE-19-Dec21-14), and the study was conducted in accordance with the Declaration of Helsinki. Blood donor eligibility followed the Saudi Central Board for Accreditation of Healthcare Institutions (CBAHI) criteria. Donors who did not complete the donation process were excluded.

### 2.2. Study Population and Ethnicity Classification

All eligible blood donors presenting during the study period were included (*n* = 10,463). Both sexes and all nationalities were eligible. Blood samples comprised 5 mL of peripheral whole blood in EDTA tubes. Donors were recruited through standard blood donation campaigns at the Riyadh Regional Blood Bank, including both walk-in donors and organized donation drives. Eligibility followed Saudi CBAHI criteria, including age 18–60 years, hemoglobin ≥12.5 g/dL, and absence of high-risk behaviors or medical conditions. All donors underwent pre-donation screening and counseling according to national protocols. Donors were classified into five ethnic categories: Saudi; Asian (including Bangladeshi, Pakistani, Indian, Indonesian, Filipino, Syrian, and other Asian nationalities); African (including Egyptian, Sudanese, Yemeni, Ethiopian, Nigerian, and other African nationalities); European; and American. Classification used a predefined scheme based on UN geoscheme definitions, applied independently by two investigators. Syrian and Yemeni donors were classified as Asian (Western Asia); Egyptian donors as African (North Africa).

### 2.3. Blood Group Typing

All samples were typed using the Immucor NEO Iris fully automated immunohematology analyzer (Immucor Inc., Norcross, GA, USA), a microplate-based robotic system employing charge-coupled device (CCD) cameras and multi-feature image analysis software. ABO forward and reverse typing, D antigen typing, and extended Rh/Kell phenotyping were performed for all 10,463 samples; one sample yielded an indeterminate D antigen result and is excluded from D+/D− analyses. Extended Rh phenotyping covered five principal antigens: D, C, c, E, and e. K antigen (KEL1) typing was included in the standard extended panel. Samples were classified into 18 Rh phenotypic combinations. Phenotypes lacking the e antigen (E+/e−) were designated rare; those bearing e were common [7].

Quality Assurance and Reaction Interpretation: The Immucor NEO system utilizes a standardized algorithm for reaction grading (0 to 4+), with automated interpretation based on cell button size and agglutination pattern. All results were reviewed by trained blood bank technologists prior to release. For samples with weak reactions (≤2+) or ambiguous results, the following confirmatory protocol was applied:

1. Repeat testing on the automated platform using fresh reagent aliquots;
2. Manual tube method confirmation using anti-D, anti-C, anti-c, anti-E, and anti-e monoclonal antisera (Bio-Rad, Hercules, CA, USA);
3. For persistently weak or discrepant D antigen results, indirect antiglobulin test (IAT) was performed to detect weak D (Du) variants;
4. Samples with unresolved serological results were referred for molecular genotyping at a reference laboratory.

During the study period, one sample (0.0096%) yielded an indeterminate D antigen result despite repeat testing and IAT; this sample was excluded from D+/D− analyses. This low rate of indeterminate results (approximately 1:10,000) is consistent with expected performance for automated solid-phase technology in a diverse donor population.

### 2.4. Statistical Analysis

Statistical analyses were performed using IBM SPSS Statistics version 25 (IBM Corp., Armonk, NY, USA). Descriptive statistics were expressed as frequencies (*n*) and percentages (%). Chi-square (χ^2^) tests were employed to evaluate associations between blood group systems and ethnicity. However, we recognize that the primary scientific contribution of this study is the descriptive frequency data, which provides essential baseline information for transfusion practice. Hypothesis testing is presented as secondary and exploratory, with the understanding that for smaller ethnic subgroups (European, American), statistical comparisons are underpowered and are presented descriptively only. To account for multiple comparisons across eight independent tests, we applied a Bonferroni-corrected significance threshold of α = 0.00625 (0.05/8). Associations meeting this threshold were considered statistically robust, while findings with *p*-values between 0.00625 and 0.05 were interpreted as exploratory and hypothesis-generating.

## 3. Results

### 3.1. Demographic Characteristics

A total of 10,463 blood donors were enrolled. The majority were male (*n* = 9962; 95.2%), with female donors comprising 4.8% (*n* = 501). Asians were the largest ethnic group (*n* = 5016; 47.9%), followed by Saudis (*n* = 3408; 32.6%), Africans (*n* = 2012; 19.2%), Europeans (*n* = 23; 0.2%), and Americans (*n* = 4; <0.1%). Top nationalities were Yemeni (13.2%), Syrian (11.2%), Egyptian (7.8%), Pakistani (5.8%), and Indian (5.6%). Demographic data are summarized in Table 1.

### 3.2. ABO Blood Group Frequency

Of 10,463 enrolled donors, 10,462 yielded classifiable ABO results (one donor had an unclassifiable result). Blood group O+ was most prevalent (*n* = 3310; 31.6%), followed by A+ (*n* = 2679; 25.6%) and B+ (*n* = 2060; 19.7%). AB+ was present in 7.8% (*n* = 817). Among Rh-negative types, O− predominated (*n* = 688; 6.6%), followed by A− (*n* = 491; 4.7%), B− (*n* = 324; 3.1%), and AB− (*n* = 93; 0.9%), the rarest type overall. Distribution is shown in Table 2.

### 3.3. Rh D Antigen and Extended Rh Phenotype Frequency

Of 10,463 samples, 10,462 yielded definitive D antigen results; full frequencies are shown in Table 3. One sample was indeterminate and is excluded from D+/D− counts (see Table 4, footnote ^†^). Of those typed, 84.7% (*n* = 8866) were D-positive, and 15.3% (*n* = 1596) were D-negative. The most prevalent Rh phenotypes were CcDee (24.2%; *n* = 2532), ccDee (19.9%; *n* = 2077), and CCDee (17.7%; *n* = 1847), all common (e-bearing) phenotypes. Among E-bearing (E+/e+) phenotypes, CcDEe was most frequent (10.6%; *n* = 1107) and ccDEe second (9.7%; *n* = 1020). e-negative (E+/e−) phenotypes included ccDEE (the most prevalent of this group at 2.5%; *n* = 257), CCDEe (0.2%; *n* = 20), CcDEE (<0.1%; *n* = 4), and CCDEE (<0.1%; *n* = 1). Among D-negative donors (*n* = 1596), leading phenotypes were Ccdee (26.9%; *n* = 430), ccdee (24.7%; *n* = 394), and CCdee (21.9%; *n* = 349).

### 3.4. Kell Antigen (K/KEL1) Frequency

Of 10,463 donors, 10,459 yielded definitive K antigen results; four samples were indeterminate and excluded. K-positivity was 13.7% (*n* = 1438) and K-negativity was 86.3% (*n* = 9021). By ethnicity: Africans (15.4%), Saudis (13.5%), Asians (13.3%), Europeans (13.0%). No American donors (*n* = 4) were K-positive, though this subgroup is underpowered and this data is descriptive only. The association between K antigen and ethnicity was not significant (χ^2^ = 7.391, df = 8, *p* = 0.495).

### 3.5. Correlation Analyses

#### 3.5.1. ABO Blood Group and K Antigen

ABO distribution was broadly similar between K-positive (*n* = 1438) and K-negative (*n* = 9021) donors. Among K-positive donors: O+ (31.2%), A+ (23.2%), B+ (21.3%), AB+ (8.3%), O− (8.2%), A− (4.5%), B− (2.4%), AB− (0.9%). No significant association was found (χ^2^ = 23.475, df = 16, *p* = 0.102).

#### 3.5.2. ABO Blood Group and D Antigen

When stratified by D antigen, blood group O was most common in both D-positive (37.3%) and D-negative donors (43.1%), while AB was least common in both (9.2% and 5.8%, respectively; Table 4). The ABO × D antigen cross-tabulation was significant (χ^2^ = 10,483.017, df = 36, *p* < 0.001), reflecting classification structure rather than a novel biological association.

#### 3.5.3. Rh Phenotype and ABO Blood Group

Rh phenotype distribution was consistent across ABO types. CcDee was dominant regardless of ABO group: 28.7% of O+, 29.4% of A+, 28.3% of B+, and 25.8% of AB+ donors. The E-bearing CcDEe phenotype was present in 12.2–12.8% across D-positive ABO groups. The significant association (*p* < 0.001) reflects classification structure and should not be interpreted as an independent biological finding.

#### 3.5.4. Rh Phenotype and Ethnicity

Rh phenotype distribution varied significantly by ethnicity (χ^2^ = 109.186, df = 72, *p* = 0.003). CcDee was the most prevalent phenotype across Saudis (22.9%), Asians (24.9%), and Africans (24.6%). European donors showed relatively higher CcDee (39.1%) and CcDEe (21.7%) frequencies, though the European group (*n* = 23) is too small for inferential conclusions. Selected frequencies are in Table 5.

#### 3.5.5. Rh Phenotype and K Antigen

Among K-positive donors, CcDee was most frequent (26.8%), followed by ccDee (17.7%) and CCDee (16.6%). The ccDEe phenotype was slightly more prevalent among K-positive (11.3%) than K-negative donors (9.5%). A statistically significant association was observed (χ^2^ = 55.620, df = 36, *p* = 0.019). However, this result did not meet the Bonferroni-corrected significance threshold (α = 0.00625) and is therefore considered exploratory and hypothesis-generating. Given that the *RHCE* and *KEL* genes reside on different chromosomes (chromosome 1 and 7, respectively), this association—if confirmed—likely reflects population stratification or founder effects rather than a biological linkage.

#### 3.5.6. D Antigen and Ethnicity

D antigen positivity varied across ethnic groups: Americans (100.0%), Europeans (95.7%), Africans (87.4%), Asians (86.1%), and Saudis (81.1%). Saudi donors had the highest D-negativity (18.9%). The association was highly significant (χ^2^ = 56.798, df = 8, *p* < 0.001; Table 6).

#### 3.5.7. ABO and Ethnicity

ABO distribution varied significantly across ethnic groups. Blood group O+ was most prevalent among Saudis (32.0%), Americans (75.0%), and Africans (34.3%), while A+ was most common among Asians (27.3%) and Europeans (30.4%) (χ^2^ = 94.181, df = 32, *p* < 0.001; Table 7).

#### 3.5.8. K Antigen and Ethnicity

K antigen positivity rates were broadly comparable: Africans (15.4%), Saudis (13.5%), Asians (13.3%), and Europeans (13.0%). The association was not significant (χ^2^ = 7.391, df = 8, *p* = 0.495). A summary of all Chi-square results is provided in Table 7.

## 4. Discussion

This study provides, to our knowledge, one of the largest ethnically stratified serological blood group datasets reported from the Riyadh region to date, characterizing ABO, Rh phenotype, and K antigen frequencies across 10,463 donors representing over 50 nationalities. While the primary contribution is the establishment of comprehensive baseline frequency data for this diverse donor population, the findings have direct implications for transfusion practice, inventory management, and the development of rare donor registries.

The overwhelming predominance of male donors (95.2%) is consistent with other Arabian Peninsula studies: a Jeddah study documented 98.3% male donors [13] and an Omani cohort reported 96.8% [14]. This contrasts with Western European settings where sex parity is typical [1]. The disparity likely reflects hemoglobin and body weight eligibility thresholds, cultural norms, and limited gender-targeted awareness campaigns, reinforcing the need for female-focused donor outreach.

Blood group O+ was the most prevalent ABO type (31.6%), consistent with data from Riyadh [15], Albaha [16], Asir [17], Aljouf [18], and Jazan [19], and aligning with global reports from Oman (50.47%) [14] and Nigeria (44.5%) [20]. Egypt and Pakistan report blood group A as most prevalent [21,22], likely reflecting distinct ABO allele frequencies. Blood group AB− was universally the rarest type (0.9%).

The D-positivity rate of 84.7% is slightly lower than from other Saudi regions (Albaha 90.88% [16]; Aljouf 91.2% [18]; Western region ~91% [23]). Saudi donors showed the lowest D-positivity by ethnicity (81.1%), consistent with prior data demonstrating higher D-negativity in Saudi nationals relative to Asian populations [24]. This likely reflects a higher frequency of the *RHD*-deletion haplotype in Arabian Peninsula populations compared to South and Southeast Asian populations—a well-recognized pattern of molecular variation in the Rh blood group system. The high D-negativity proportion (18.9%) among Saudi donors has significant clinical implications: nearly one in five is D-negative, making mandatory D typing and D-negative blood reservation essential components of transfusion safety policy.

The most prevalent Rh phenotype was CcDee (24.2%), followed by ccDee (19.9%) and CCDee (17.7%), broadly consistent with Elsayid et al., who reported DCcee (equivalent to CcDee in Fisher-Race notation) as most common in Riyadh [15]. The Aljouf study found CcDEe as the leading phenotype [25], but enrolled only male Arab donors from five specific tribes, substantially limiting representativeness. The relatively high prevalence of the E-bearing CcDEe phenotype (10.6%) underscores the value of extended Rh phenotyping in diverse donor populations.

The K antigen positivity of 13.7% closely matches the 14% reported by Alalshaikh et al. from Riyadh [26], and exceeds rates from southwestern Saudi Arabia [27], likely reflecting Riyadh’s greater ethnic diversity. Africans showed the highest K-positivity (15.4%), consistent with Egyptian data reporting 23.6% [21]. The absence of a significant K antigen–ethnicity association (*p* = 0.495) may reflect the population-diluting effect of the large, multi-ethnic donor base.

The association between Rh phenotype and K antigen (*p* = 0.019) did not survive the Bonferroni-corrected threshold (α = 0.00625) and must be interpreted as exploratory. Notably, the *RHCE* and *KEL* genes reside on different chromosomes (chromosomes 1 and 7, respectively), making inter-locus linkage disequilibrium biologically implausible. Consequently, we suggest that this association, if confirmed in future studies, may be a result of population stratification or founder effects within the donor pool rather than a novel genetic linkage. Future molecular studies are required to confirm or refute these observations, and we caution against any clinical or biological interpretation until such confirmatory data are available.

The significant associations between Rh phenotype and ethnicity (*p* = 0.003) and D antigen and ethnicity (*p* < 0.001) confirm that blood group antigen frequencies are population-stratified in the Riyadh donor pool. For patients with sickle cell disease and thalassemia requiring chronic transfusions, extended phenotype matching reduces alloimmunization risk substantially [8].

While the current study provides comprehensive serological baseline data, future investigations incorporating molecular blood group genotyping—particularly for RhD variants and weakly expressed antigens—will be essential to fully characterize the genetic architecture underlying these phenotypic distributions. Similarly, prospective studies examining the Rh phenotype distribution among transfusion recipients, especially those with hemoglobinopathies, would directly inform extended matching protocols.

Several limitations warrant acknowledgment. The retrospective design precludes control over all confounding variables. Detailed information about donor recruitment processes was not available for all variables, and patient Rh distribution data were not collected. The predominantly male cohort limits generalizability, particularly for female patients in whom K antigen implications are greatest. European (*n* = 23) and American (*n* = 4) subgroups are too small for inferential purposes. Serological phenotyping is susceptible to weak antigen expression; molecular genotyping would provide greater precision. Future studies incorporating molecular blood group genotyping, expanded antigen panels (Duffy, Kidd, MNS), and female-specific analyses are strongly recommended.

## 5. Conclusions

This large-scale retrospective study provides comprehensive, ethnically stratified baseline data on ABO blood group, Rh phenotype, and K antigen frequencies among blood donors at the Riyadh Regional Blood Bank—one of the largest reported datasets of its kind from this region, to our knowledge [15,26]. Blood group O+ was the most prevalent ABO type (31.6%), CcDee the most common Rh phenotype (24.2%), and K antigen positivity was 13.7%. The high Rh D-negativity among Saudi donors (18.9%)—substantially higher than the rate in Asian and African donors—has immediate implications for D-negative blood inventory management and alloimmunization prevention.

Statistically significant associations between D antigen and ethnicity (*p* < 0.001) and ABO and ethnicity (*p* < 0.001) were robust to Bonferroni correction and confirm population-stratified antigen frequencies in this diverse urban setting. The association between Rh phenotype and K antigen (*p* = 0.019) did not survive Bonferroni correction and should be considered hypothesis-generating; while exploratory, it warrants further molecular investigation given that inter-locus linkage disequilibrium between *RHCE* (chromosome 1) and *KEL* (chromosome 7) is not a biologically plausible mechanism. This finding requires molecular confirmation before any biological or clinical conclusions can be drawn. The association between Rh phenotype and ethnicity (*p* = 0.003) met the Bonferroni-corrected threshold (α = 0.00625) and is considered statistically robust.

These findings support proportional blood type inventory planning, evidence-based extended phenotyping protocols for high-risk patients—including those with sickle cell disease and thalassemia requiring repeated transfusions—and the data-informed development of a rare donor registry through targeted ethnicity-based recruitment. Sustained investment in donor awareness campaigns—particularly those directed at female and Saudi donors—alongside the systematic adoption of extended phenotyping, will further strengthen transfusion safety in Riyadh.

## Figures and Tables

**Table 1:** Demographic characteristics of blood donors (*n* = 10,463).

Characteristic	Category	*n*	%
Sex	Male	9962	95.2
	Female	501	4.8
Ethnicity	Asian	5016	47.9
	Saudi	3408	32.6
	African	2012	19.2
	European ^†^	23	0.2
	American ^†^	4	<0.1

^†^ European and American subgroups have very small sample sizes (*n* = 23 and *n* = 4, respectively); all percentages and statistical comparisons involving these groups are descriptive only and should not be interpreted inferentially.

**Table 2:** Frequency of ABO blood groups (*n* = 10,462 classifiable).

Blood Group	*n*	%
O+	3310	31.6
A+	2679	25.6
B+	2060	19.7
AB+	817	7.8
O−	688	6.6
A−	491	4.7
B−	324	3.1
AB−	93	0.9
Total *	10,462	99.9

* One donor had an unclassifiable ABO result and is excluded (enrolled cohort *n* = 10,463).

**Table 3:** Frequency of Rh phenotypes among blood donors (*n* = 10,463).

Rh Phenotype	*n*	%	Category
CcDee	2532	24.2	Common
ccDee	2077	19.9	Common
CCDee	1847	17.7	Common
CcDEe	1107	10.6	E-bearing (E+/e+)
ccDEe	1020	9.7	E-bearing (E+/e+)
Ccdee	430	4.1	D-negative
ccdee	394	3.8	D-negative
CCdee	349	3.3	D-negative
ccDEE	257	2.5	Rare (E+/e−)
ccdEe	190	1.8	D-negative
CcdEe	178	1.7	D-negative
ccdEE	47	0.4	D-negative
CCDEe	20	0.2	Rare (E+/e−)
CCdEe	7	0.1	D-negative
CcDEE	4	<0.1	Rare (E+/e−)
CCdEE	1	<0.1	D-negative
CcdEE	1	<0.1	D-negative
CCDEE	1	<0.1	Rare (E+/e−)
Total	10,463	100.0	

**Table 4:** Distribution of ABO blood groups by D antigen status.

ABO Group	D-Positive (*n*)	D-Positive (%)	D-Negative (*n*)	D-Negative (%)	Total
O	3310	37.3	688	43.1	3998
A	2679	30.2	491	30.8	3170
B	2060	23.2	324	20.3	2384
AB	817	9.2	93	5.8	910
Total	8866	84.7	1596	15.3	10,462

One donor had an indeterminate D antigen result and is excluded from D+/D− counts; enrolled cohort *n* = 10,463.

**Table 5:** Selected Rh phenotype frequencies by ethnicity.

Rh Phenotype	Saudi (%)	Asian (%)	African (%)	European (%) ^†^	American (%) ^†^
CcDee	22.9	24.9	24.6	39.1	0.0
ccDee	18.6	20.5	20.5	4.3	0.0
CCDee	17.2	17.1	19.6	30.4	75.0
CcDEe *	10.1	10.9	10.6	21.7	0.0
ccDEe *	9.6	10.2	9.0	0.0	0.0
Ccdee	5.0	3.6	3.8	0.0	0.0
ccdee	4.8	3.6	2.4	4.3	0.0

* E-bearing phenotype (E+/e+). ^†^ European (*n* = 23) and American (*n* = 4) groups are too small for inferential interpretation; descriptive only.

**Table 6:** D antigen positivity by ethnicity.

Ethnicity	D-Positive (*n*)	D-Positive (%)	D-Negative (*n*)	D-Negative (%)	Total
**Saudi**	2764	81.1	644	18.9	3408
**Asian ^‡^**	4318	86.1	697	13.9	5016
**African**	1758	87.4	254	12.6	2012
**European ^†^**	22	95.7	1	4.3	23
**American ^†^**	4	100.0	0	0.0	4
**Total**	8866	84.7	1596	15.3	10,463

^†^ Small sample sizes; descriptive only. ^‡^ One Asian donor had an indeterminate D result; D+/D− counts sum to 5015. *p* < 0.001 (χ^2^ = 56.798, df = 8).

**Table 7:** Summary of Chi-square association tests.

Association	χ^2^ Value	df	*p*-Value
ABO × K antigen	23.475	16	0.102 (NS)
Rh phenotype × D antigen *	10,483.017	36 ^†^	<0.001
Rh phenotype × ethnicity	109.186	72	0.003
D antigen × ethnicity	56.798	8	<0.001
K antigen × ethnicity	7.391	8	0.495 (NS)
Rh phenotype × K antigen	55.620	36	0.019 ^‡^
ABO × ethnicity	94.181	32 ^§^	<0.001

NS = not significant. * Structurally constrained—association reflects classification structure (Rh phenotypes inherently include D antigen status), not a biologically independent finding. This analysis is included for completeness only and is not interpreted in the Discussion. ^†^ df = 36: (19 − 1) × (3 − 1) = 36; the contingency table included 19 phenotype rows as classified by SPSS, comprising 18 established phenotypes and one single-donor data-entry variant (ccDEc). ^‡^ Did not meet Bonferroni-corrected threshold (α = 0.00625); considered exploratory. ^§^ df = 32: (9 − 1) × (5 − 1) = 32.

## Data Availability

The data are available from the corresponding author upon reasonable request and with permission from the Riyadh Regional Blood Bank.

## Abbreviations

The following abbreviations are used in this manuscript:

**ABO**	ABO blood group system
**CBAHI**	Saudi Central Board for Accreditation of Healthcare Institutions
**EDTA**	Ethylenediaminetetraacetic acid
**HDFN**	Hemolytic disease of the fetus and newborn
**IRB**	Institutional Review Board
**ISBT**	International Society of Blood Transfusion
**KEL/K**	Kell blood group system/K antigen (KEL1)
**RBC**	Red blood cell
**Rh**	Rhesus blood group system
**SPSS**	Statistical Package for the Social Sciences
